# Neuronal Surface Autoantibodies in Neuropsychiatric Disorders: Are There Implications for Depression?

**DOI:** 10.3389/fimmu.2017.00752

**Published:** 2017-07-05

**Authors:** Shenghua Zong, Carolin Hoffmann, Marina Mané-Damas, Peter Molenaar, Mario Losen, Pilar Martinez-Martinez

**Affiliations:** ^1^Division Neuroscience, School for Mental Health and Neuroscience, Maastricht University, Maastricht, Netherlands

**Keywords:** neuronal surface autoantibodies, neuropsychiatric disorders, depression, pathogenicity, immunoglobulin, neurotransmitter receptor, ion channel, blood–brain barrier

## Abstract

Autoimmune diseases are affecting around 7.6–9.4% of the general population. A number of central nervous system disorders, including encephalitis and severe psychiatric disorders, have been demonstrated to associate with specific neuronal surface autoantibodies (NSAbs). It has become clear that specific autoantibodies targeting neuronal surface antigens and ion channels could cause severe mental disturbances. A number of studies have focused or are currently investigating the presence of autoantibodies in specific mental conditions such as schizophrenia and bipolar disorders. However, less is known about other conditions such as depression. Depression is a psychiatric disorder with complex etiology and pathogenesis. The diagnosis criteria of depression are largely based on symptoms but not on the origin of the disease. The question which arises is whether in a subgroup of patients with depression, the symptoms might be caused by autoantibodies targeting membrane-associated antigens. Here, we describe how autoantibodies targeting membrane proteins and ion channels cause pathological effects. We discuss the physiology of these antigens and their role in relation to depression. Finally, we summarize a number of studies detecting NSAbs with a special focus on cohorts that include depression diagnosis and/or show depressive symptoms.

## Introduction

Neuronal surface autoantibodies (NSAbs) have been described mainly in autoimmune encephalitis, a group of newly defined neuroimmunological disorders (1). Those autoantibodies target essential neurotransmitter receptors, ion channels, or associated proteins on the membrane of neuronal cells, such as *N*-methyl-d-aspartate receptor (NMDAR) (2), α-amino-3-hydroxy-5-methyl-4-isoxazolepropionic acid receptor (AMPAR) (3, 4), metabotropic glutamate receptor 1 (mGluR1) (5), metabotropic glutamate receptor 5 (mGluR5) (6), GABA_B_ receptor (GABA_B_R) (7), GABA_A_ receptor (GABA_A_R) (8–10), leucine-rich, glioma inactivated 1 (LGI1) and contactin-associated protein-like 2 (Caspr2) (11), dipeptidyl aminopeptidase-like protein 6 (DPPX) (12–14), and dopamine receptor D_2_ (D2R) (15). Antibody-positive cases are associated with a spectrum of neurological disorders including limbic encephalitis, neuromyotonia, Morvan’s syndrome, epilepsy, and psychiatric disorders (16–19).

Depression is a psychiatric disorder with complex etiology and pathogenesis. The International Classification of Diseases and The Diagnostic and Statistical Manual of Mental Disorders are widely used for the diagnoses of this disorder, based on symptoms but not on the cause of the disease. There are several theories about the causes of depression and immune dysregulation is one of them. The relationship between the immune system and depression has been widely discussed. To date, most research has focused on pro-inflammatory cytokines and a few reviews also propose a direct link between autoantibodies and depression (20, 21). Studies investigating the presence of autoantibodies in depression have focused in those targeting peripheral organs like the thyroid and intracellular antigens such as antinuclear antibodies and ribosomal-P antibodies (21–25). During the past decade, it has become clear that NSAbs could cause severe neuropsychiatric disorders. Since some of the NSAbs interfere with neurotransmission pathways related to depression (26–28), a subtype of depression may be caused by antibody-mediated autoimmunity and, therefore, might potentially respond to immunotherapy. In the current review, we summarize the literature about NSAbs in autoimmune encephalitis and psychiatric disorders, with a special focus on what is known regarding NSAbs in depression, evaluate the techniques used and how results can be interpreted, and identify research gaps. Together, we aim to provide insight into the potential role of NSAbs in depression based on the function of relevant neurotransmitter receptors and ion channels as well as autoantibody effector mechanisms.

## How NSAbs Reach the Central Nerves System (CNS)

Because neuronal surface proteins are the target of the autoantibodies discussed in this review, it is important to first understand how those autoantibodies get access to the CNS. Now it is widely accepted that the CNS is targeted by the immune system, yet the mechanism how autoantibodies go through the blood–brain barrier (BBB) is still unclear. Under normal conditions, immunoglobulins go through the BBB at a very low rate; a good example is immunoglobulin G (IgG). IgG concentration in the cerebrospinal fluid (CSF) is approximately 1% of the levels in the peripheral circulation (29–31). This indicates that once the autoantibodies reach the CNS they can cause disease as it has been observed in autoimmune encephalitis. In certain situations, like inflammation, for example, during the group A Streptococcus infection, specific Th17 cells could migrate into the brain through the cribriform plate along olfactory sensory axons. The Th17 cells expressed IL-17A which induced endothelial tight junction breakdown, increasing BBB permeability and facilitating the penetration of IgG in the brain (32). Additionally, the BBB may become leaky because of stroke, brain trauma, hemorrhages, microangiopathy, or brain tumors, and antibody penetration rate might increase. In this regard, a study has reported that autoantibodies to NMDAR (anti-NMDAR) seropositive schizophrenia patients with a history of neurotrauma or birth complications had more severe neurological symptoms than seronegative patients. And intravenous injections of extracted Ig fractions (IgG, IgA, or IgM) from anti-NMDAR seropositive patients to BBB leaky (ApoE−/−) mice could induce a psychosis-related response (33). A further study confirmed that APOE4 carrier status and anti-NMDAR seropositivity together were significantly associated with schizoaffective disorder (34). Those results indicate the importance of the BBB for anti-NMDAR-mediated pathology.

Besides, intrathecal synthesis is another possible source for autoantibodies in the CNS. B-cells can migrate to the brain and produce autoantibodies locally (35–37). This is also important to keep in mind when thinking about therapy because any potential drug against B cells has to pass the BBB to be effective. The evidence is mainly from studies analyzing autoantibodies in serum and CSF from encephalitis patients. It has been reported that in some encephalitis patients, autoantibodies targeting the NMDAR, AMPAR, GABA_B_R, DPPX, mGluR1, or mGluR5 were found only in the CSF (38). A postmortem study showed the presence of CD138+ plasma cells in the brain of NMDAR encephalitis patients, suggesting intrathecal synthesis of autoantibodies (36). Intrathecal antibody synthesis was also described in a case with autoantibodies against the mGluR1 where the patient did not respond to immunotherapy, while serum antibody levels dropped but CSF levels were still high (39). Other NSAbs, such as autoantibodies to LGI1, Caspr2, glycine receptor, and GABA_A_R may, in rare instances, be identified only in serum but be absent in CSF (38). However, if the autoantibodies are immunoabsorbed by the antigen in the brain, they might still have effects and play a pathogenic role even they are not detectable in the CSF (40).

## IgG Effector Functions

**Figure 1:**
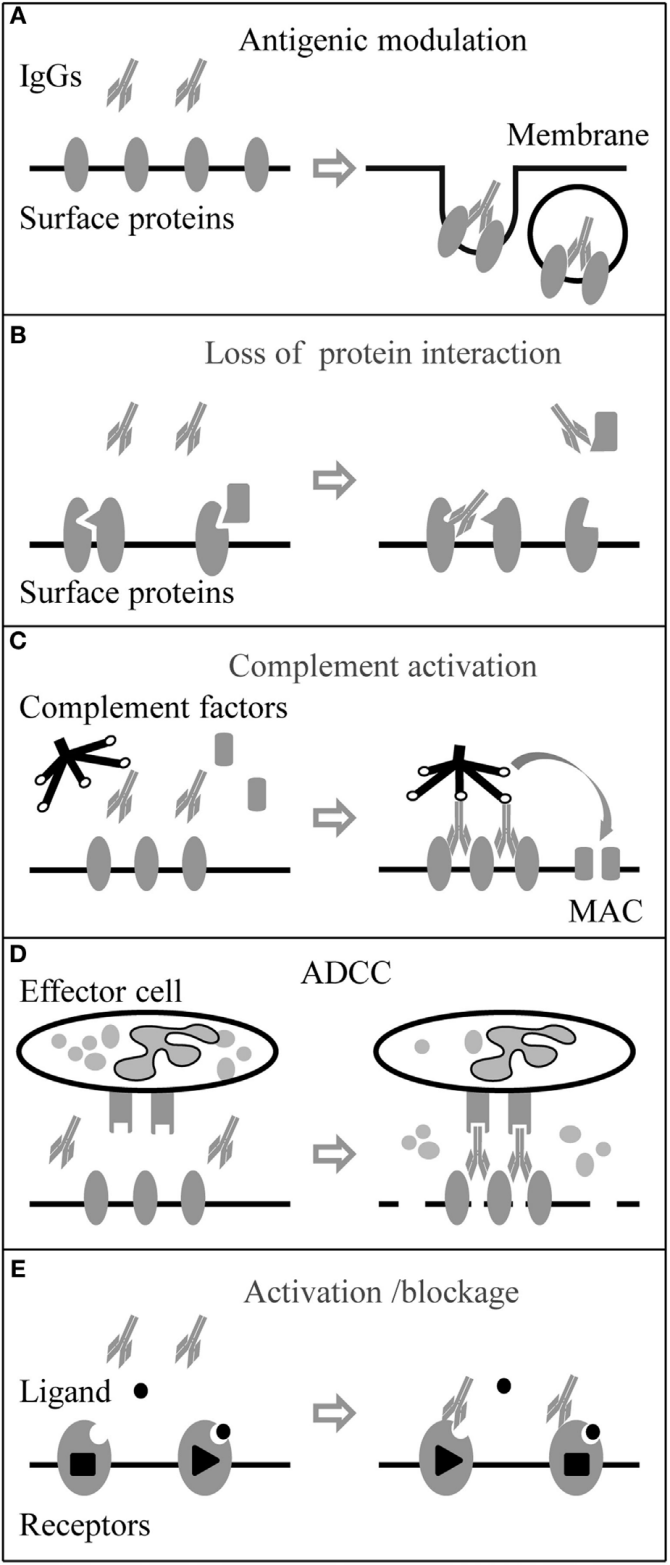
Immunoglobulin G (IgG) autoantibody effector mechanisms. Neuronal surface proteins like G-protein coupled receptors, ion channels, and associated proteins can be the targets of autoantibodies. **(A)** Autoantibodies can directly target surface proteins and induce their internalization by cross-linking of the antigens. **(B)** Autoantibodies can also target associate proteins and block protein–protein interaction. **(C)** Autoantibodies (IgG3 > IgG1 > IgG2) can activate the complement system and form the membrane attack complex (MAC) leading to damage of the membrane. **(D)** Autoantibodies binding to effector cell with Fc receptors (FcRs) can trigger antibody-dependent cell-mediated cytotoxicity (ADCC). **(E)** In addition, autoantibodies can be agonists or antagonists and activate or block the function of membrane receptors.

Antibodies (or Igs) are produced by plasma B cells. They are defined as IgM, IgG, IgA, IgD, and IgE isotypes according to heavy chain C domains. Different types of NSAbs (IgM, IgA, and IgG) have been found so far; IgG autoantibodies are considered the most pathogenic (1, 10, 33). IgG, composed of two paired heavy chain and light chain, is the major antibody in body fluid and a crucial player in the humoral immune response. In humans, four IgG isotypes (IgG1–4) exist, which have different ability to activate the complement system (41). IgG1–3 mediate pro-inflammatory activities, while IgG4 also has anti-inflammatory activities (42). The functions of IgG effector in myasthenia gravis (MG) and other well-studied autoimmune disorders are schematically illustrated in Figure 1.

### Antigenic Modulation

Antibodies of the IgG1–3 subtypes are able to cross-link the antigens because of their bivalent nature, whereas the IgG4 subtype loses this ability after the fab-arm exchange with other unrelated IgG4 molecules (43). Cross-linking autoantibodies are believed to bring the antigens close together on the cell membrane and promote the degradation of the ligand–receptor complex (44). In the case of MG, antiacetylcholine receptor autoantibodies (anti-AChR), mainly IgG1 and IgG3, are able to cross-link adjacent AChR molecules, leading to rapid internalization by endocytosis and AChR degradation (45, 46). Previous studies indicated that anti-NMDAR, IgG1–3, led to a reduction in the synaptic and extrasynaptic receptors and further decreased the synaptic plasticity and transmission (47–50). Anti-GABA_A_R, IgG1 and IgG3, had a similar effect with a reduction of GABA_A_R clusters in both synaptic and extrasynaptic areas (8–10). Also, application of anti-AMPAR (GluR1/2) to neuronal cultures significantly decreased the number of AMPAR clusters at synaptic and extrasynaptic areas by increasing the internalization of AMPAR clusters; the IgG subclasses were not analyzed in these studies (4, 51).

### Complement Activation

IgG1–3 can activate the complement system by forming the membrane attack complex (MAC) and leading to membrane damage of targeted cells. Still in MG, anti-AChR binding to AChRs, which are densely packed in the folds of the postsynaptic membrane of the neuromuscular junction, results in a very high density of AChR-bound autoantibodies and hence a very tightly packed Fc region. The complement system is activated with high efficiency and as a result, MAC is formed in the postsynaptic membrane. Together with antigenic modulation, complement activation causes severe endplate membrane damage (45, 52). Brain biopsy findings support that complement activation and MAC deposition happen associated with acute neuronal cell death in anti-voltage-gated potassium channel (VGKC) complex encephalitis and Rasmussen’s encephalitis (53, 54).

### Antibody-Dependent Cell-Mediated Cytotoxicity (ADCC)

Antibody-dependent cell-mediated cytotoxicity is the process when cytotoxic effector cells of the immune system kill the antibody targeted cell by the releasing cytotoxic granules or by expressing cell death-inducing molecules. The process is activated when the Fc receptors (FcRs) on the effector cell surface bind to Fc region of target-bound antibodies (IgG, IgA, or IgE subtypes). Those effector cells include natural killer cells, monocytes, macrophages, neutrophils, eosinophils, and dendritic cells. In humans, the IgG1 subtype has the ability to strongly trigger ADCC and is used widely in therapy for certain types of cancer (55, 56). Neuromyelitis optica (NMO) is a severe inflammatory demyelinating disease in CNS, and autoantibodies against aquaporin-4 (anti-AQP4), a water channel on astrocyte play a role in the pathology of NMO by triggering complement activation and ADCC (57). *In vitro*, NMO patient serum and CSF IgG induced ADCC of glial cells transfected with AQP4 (58). *In vivo*, injection of anti-AQP4 produced large NMO lesions in mice, with the loss of AQP4 and GFAP immunoreactivity, inflammation, and demyelination. Those pathologies were largely reduced when FcγIII receptor deficient mice were used or when normal mice were injected with Fcγ receptor blocking antibody (59).

### Loss of Receptor or Ion Channel-Associated Proteins

Autoantibodies can target receptor or ion channel-associated proteins. As a result, the protein–protein interaction between the receptor and the associated protein is interrupted with the consequence that those receptors or ion channels become dysfunctional. Autoantibodies to muscle-specific kinase (anti-MuSK) are another type of autoantibodies involved in the pathogenicity of MG. Anti-MuSK (predominant IgG4) binds to an extracellular epitope on MuSK at the neuromuscular junction, inhibits the pathway involved in the clustering of the AChRs in the membrane, and leads to failure of neuromuscular transmission (43). Autoantibodies to LGI1, a VGKC complex-associated protein, play a similar role, resulting in reduced VGKC function at CNS synapses and increased cell excitability (60). Besides, anti-LGI1 also interferes with other surface receptors. LGI1 interacts with the ADAM22/23, epilepsy-related transmembrane proteins, and regulates AMPAR-mediated synaptic transmission in the hippocampus (61, 62). Additionally, an *in vitro* study showed that anti-LGI1 from encephalitis patients blocked the binding of LGI1 to ADAM22 by neutralizing the ADAM22-binding domain of LGI1. The loss of LGI1-ADAM22 interaction could further reduce synaptic AMPAR, which indirectly associates with ADAM22 (63). Importantly, this indicates that besides their direct effect on ion channel/receptors, autoantibodies may interfere with protein–protein interaction and have consequences for synapse formation, function, and maintenance.

### Activation, Inactivation, and Functional Receptor Blockage of the Receptors

Autoantibodies may activate, inactivate, or block ion channels and neurotransmitter G protein-coupled receptors (64). Serum IgG from MG patients has been shown to block the ACh binding sites in cultured mammalian muscle cells (65) and caused acute and severe muscle weakness in rodents, independent of inflammation or necrosis (66). Autoantibodies against the γ subunit of the AChR which is only present in embryonic forms of the receptor have been reported in some cases to block the AChR function and cause arthrogryposis multiplex congenita (67). Conversely, AChR antibodies can also induce prolonged open time of the AChR leading to muscle weakness by excitotoxicity at the neuromuscular junction (68). Anti-AMPAR (GluR3B subunit) autoantibodies (anti-AMPA-GluR3B) can activate AMPAR that contains the GluR3B subunit, leading to the spontaneous occurrence of ion currents (69, 70). In an animal study, anti-AMPA-GluR3B produced following immunization with the GluR3B peptide bonded cultured neurons, evoked GluR ion channel activity, and killed neurons by “excitotoxicity” (71). When autoantibodies target G-protein-coupled receptors, they can interfere with signaling pathways, which might lead to slow effector responses. An example is Graves’ disease, where autoantibodies against the thyroid-stimulating hormone (TSH) receptor stimulate the synthesis of thyroid hormone, which is produced in excess and results in hyperthyroidism. Additionally, there are anti-TSH receptor antibodies that block the signal transduction and consequently reduce thyroid hormone production by targeting different epitopes of the receptor (72).

## The Targets of NSAbs are Relevant in the Pathology of Depression

Monoamine imbalance is the main biochemical postulate of depression. Both serotonergic neurotransmission and dopaminergic neurotransmission play important roles in causing depressive symptoms (73). Genetic studies suggest that polymorphisms within genes that encode for 1A serotonin receptor (5-HT1A) and D4 dopamine receptor, increase the risk of major depressive disorder (MDD) (74). 5-HT1A (75, 76) and D2DR (77, 78) levels are decreased in this disorder and both are the targets of several antidepressants (79).

Increasing evidence supports that glutamatergic and GABAergic systems are also involved in depression (27, 28). Glutamate is the predominant excitatory neurotransmitter in the CNS (80, 81). Blockade of glutamate uptake from the synapse has been reported to reduce sensitivity to reward, a symptom of depression (82). Ketamine and other NMDAR antagonists have antidepressant effects (83). Antidepressants such as imipramine can enhance the synaptic expression of GluR1, a subunit of AMPAR (84).

Interestingly, GABA concentration is reduced in cortical brain and CSF in MDD and this deficit could be reversed by chronic treatment with selective serotonin reuptake inhibitors and electroconvulsive therapy (85–87). Studies reported that cortical GABA(A)-benzodiazepine receptor complex affinity and/or number were reduced in MDD. Additionally, mice heterozygous for the γ2 subunit of GABA_A_R (γ2+/−) exhibited anxious–depressive behavior (88, 89). In this model, GABA_A_R numbers were unaltered, but had reduced benzodiazepine binding sites.

**Figure 2:**
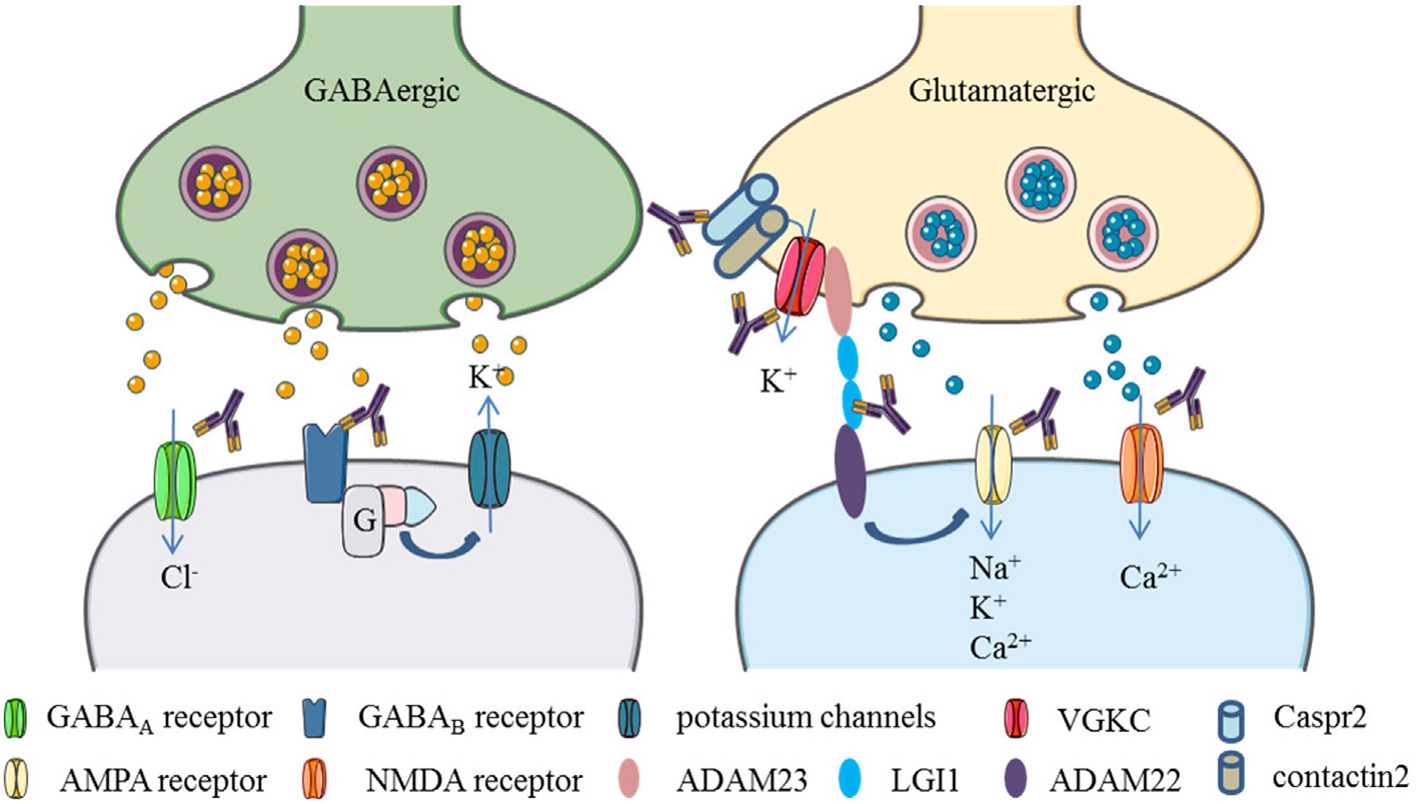
Neuronal surface autoantibodies target neuronal receptors, ion channels, and/or associate proteins that commonly affect GABA and glutamate transmission in the brain. (1) GABA receptor activation causes chloride anions influx and potassium flow-out, resulting in the hyperpolarization of the postsynaptic neurons. Autoantibodies to GABA_A_ or GABA_B_ receptors cause internalization of those membrane proteins and block the GABA transmission, leading to excitation of the postsynaptic neurons. (2) Glutamate receptors activation causes polarization of the postsynaptic neurons by positive ions (Ca2+, Na+, K+) influx. Autoantibodies to NMDA and AMPA receptors drive internalization of those receptors and block the glutamate transmission. (3) Potassium channels can be activated by GABA_B_ receptors through G proteins. Some proteins like leucine-rich, glioma inactivated 1 (LGI1) and contactin-associated protein-like 2 (Caspr2), contactin 2, ADAM22, and ADAM23 are associated with voltage-gated potassium channels (VGKCs). LGI1 can enhance AMPA receptor-mediated synaptic transmission by binding to ADAM22. Autoantibodies target to those associate proteins would cause VGKCs or AMPA receptor dysfunction (Elements are partly adapted from Servier Medical Art. http://smart.servier.com/).

Thus, if the abovementioned neurotransmitter receptors or relevant proteins are targeted by autoantibodies, including ion channels and associated proteins, they could potentially cause depression-like symptoms. Below, we summarize NSAbs that target antigens which are relevant in the pathology of depression (for an illustration see Figure 2).

## Evidence of NSAbs in Depression

### Anti-Glutamate Receptor Autoantibodies

#### Anti-NMDAR

The NMDAR, as an ionotropic glutamate receptor, contains two GluN1 and two GluN2 (A–D) subunits (alternatively called NR1 and NR2) forming heterotetramers. The subunit GluN2 can be replaced by the GluN3 (A/B) subunit, which has an inhibitory effect on receptor activity (90, 91). NMDAR has a variety of physiological roles and any dysfunctions, either enhanced or decreased activity, may result in neuropsychiatric disorders such as schizophrenia, bipolar disorder, MDD, substance-induced psychosis, Huntington’s disease, Alzheimer’s disease, and neuropsychiatric systemic lupus erythematosus (NPSLE) (92). In addition, higher gene expression levels of NR1 and NR2 (A–D) are detected in female patients with MDD (93). Prolonged inhibition of the NMDAR by phencyclidine leads to memory loss, thought disorder, depression, and personality changes (94). Antagonists of the NMDAR like ketamine also have rapid antidepressant effects (95, 96). All in all, these studies suggest that NMDAR plays a critical role in psychiatric disorders including depression.

Anti-NMDARs in autoimmune encephalitis were first described in three patients with ovarian teratoma and commonly presenting with psychiatric symptoms followed by neurological manifestations including seizures, movement disorder, and dysfunction of the autonomous nervous system (2). The methods used for detection were immunohistochemistry (IHC) on rat brain tissues, immunocytochemistry on live hippocampal neurons, and fixed cell-based assay (CBA). The autoantibodies identified were present both in CSF and serum. Later studies revealed that the extracellular N-terminal domain of the NR1 subunit is the main epitope of those autoantibodies (97). A case series showed that in more than two-thirds of cases with NMDAR encephalitis patients were initially seen by psychiatrists or admitted to psychiatric centers because they showed prominent psychiatric symptoms including anxiety, agitation, bizarre behavior, delusional or paranoid thoughts, and visual or auditory hallucinations (98). Consequently, researchers broaden the search for anti-NMDAR to psychiatric disorders, mainly first episode psychosis. Bipolar and MDDs were usually included as psychiatric disorder controls. One meta-analysis indicated higher odds of anti-NMDAR in psychotic and affective disorders (99). An affective disorder cohort consisting of 148 patients was screened for anti-NMDAR, in which 24 (16.2%) were seropositive (5 were IgG, 15 IgA, and 7 IgM). The prevalence in this cohort was higher than in healthy controls (10.8%) (34). In this study, the method used was fixed CBA and the dilution of serum used was from 1 in 10 and titers for positive cases were double-determined in two laboratories. The results have been criticized because of the much higher prevalence of anti-NMDAR in healthy control than in other groups’ study results (34, 100, 101). Further complementary investigations, using a dilution of 1:320, identified a lower percentage of positive individuals in a cohort of depression patients. Anti-NMDAR (IgG, IgA, and IgM) were found to be 4.1% in depression, still higher than healthy control (1.7%) at the significant level (33, 99). The author explained the increased number of seropositive anti-NMDAR cases in affective disorder cohort by the fact that the mean age of the affective disorder group was higher than in the control group (autoantibody prevalence is generally increasing with age) (33). Another study using same methods found 10.6% (1.9% IgG) positive for anti-NMDAR affective disorder cohort (*n* = 310) but no significant difference for healthy control (102). Additionally, another study analyzed a depression cohort (*n* = 70) and found two (2.9%) seropositive patients for NMDAR (both IgA) and one seropositive (0.4%) (IgM) result in a healthy control (*n* = 230), so none of them were IgG (101). The experiment was replicated and higher numbers of seropositive cases were found both in healthy controls and the disease groups (103). Early studies by Dickerson et al. (104) (ELISA, peptide of NR2, *n* = 28) and Zandi et al. (105) using variations of the methodology (live CBA) did not report any positive results in depression cohorts. Passive transfer of anti-NMDAR (NR1) to mice could cause depressive-like symptoms (106). However, the correlation of symptoms in animal models with those observed in humans needs to be further demonstrated (107).

In contrast to anti-NMDAR in autoimmune encephalitis which mainly targets the NR1 subunit, Lapteva and colleagues found that autoantibodies targeting the NR2 subunit of NMDAR were associated with depression in systemic lupus erythematosus (SLE) patients (108). In fact, anti-NR2A/B autoantibodies were thought to be a subset of the anti-double-stranded DNA (dsDNA) antibodies (109). The epitope identified to be targeted by the antibodies in this study was a pentapeptide Asp/Glu-Trp-Asp/Glu-Tyr-Ser/Gly. This sequence present on the NR2A/B subunit is a mimotope of anti-dsDNA. This was confirmed by showing that affinity-purified antibodies from SLE patients targeting this peptide also bind to dsDNA (109, 110). Moreover, those autoantibodies mediated apoptotic death of neurons *in vivo* and *in vitro* (109). Several studies have investigated the role of anti-NR2 in NPSLE and found that the antibody may lead to dysfunction of NMDAR *in vitro* and that passive transfer of anti-NR2 in animals induced neuronal apoptosis and affects animal memory and cognitive ability (111, 112).

Anti-NMDAR autoantibodies in depression are still questionable since most of these studies considered the depression cohorts as control groups and numbers were relatively small. Variations in the methodology make it difficult to compare results from different groups, which is a common fact that should be kept in mind through this review. In particular, the methodology varies among studies (CBA or ELISA), or the same methodology is used with different experimental conditions (fixed or live CBA) by different groups, different subunits of the antigens are employed (NR1, NR1, and NR2a/b together in CBA, NR2 peptide in ELISA), different body fluids (serum, plasma, or CSF), different immunoglobulins detected (IgG, IgA, and/or IgM) and different dilutions of the sample used (from 1:10 to 1:320) (17).

#### Anti-AMPAR

AMPAR is another ionotropic glutamate receptor which mediates the fast excitatory neurotransmission in the CNS (113). The majority of AMPAR are tetramers composed of two GluR2 and either two GluR1, three, or four subunits that combine in a brain region-dependent manner (114, 115). GluR1/2 and GluR2/3 receptors are highly expressed in the synaptic CA3-CA1 areas of the hippocampus. Besides, they are also expressed in cerebellum and caudate putamen (116).

Lai and colleagues first reported autoantibodies to AMPAR (GluR1 and GluR2 subunits) in limbic encephalitis (4). The clinical features of this type of autoimmune encephalitis are short-term memory deficits, emotional/behavioral changes, and seizures, frequent association with paraneoplastic disease, treatment responsiveness and has a tendency to relapse (4). GluR3 has been identified as an autoantigen in Rasmussen’s encephalitis in which the clinical characteristics of these patients were mainly epilepsy and language problems (117, 118). An anti-AMPAR (GluR1)-positive case was reported with breast ductal infiltrating adenocarcinoma that showed behavioral changes, depressed mood, and memory loss during the process of the disease without seizures (3). In contrast, screening for anti-AMPAR (GluR1 and GluR2) in a depression cohort (*n* < 380) by fixed CBA using 1:10 diluted serum did not report any positive cases (101, 102).

### Anti-GABA Receptor Autoantibodies

#### Anti-GABA_A_ Receptor

GABA_A_R are ionotropic receptors and GABA is the ligand. There are several subunit isoforms (α, β, and γ) for the GABA_A_R, which determine the receptor’s agonist affinity, chance of opening, conductance, and other properties. Subunits of GABA_A_R have a different distribution in the brain and may respond with a different sensitivity to GABA, leading to a different function. A decline in GABA_A_R signaling triggers hyperactivity in neurological disorders such as insomnia, anxiety, and epilepsy.

Autoantibodies to GABA_A_R were recently identified in autoimmune encephalitis. The clinical features varied in different studies. Petit-Pedrol et al. reported a series of 18 patients with anti-GABA_A_R, of whom 6 had high titer antibodies detected both in blood and CSF and showed severe encephalitis and refractory seizures (8). The other 12 patients with lower titers in serum had different diagnoses. Six showed encephalitis with seizures, four had stiff-person syndrome, and two had opsoclonus-myoclonus. Anti-GABA_A_R in lower titers was also found in 5 of these 12. The autoantibodies targeted α1 and β3 subunits and caused selective reduction of the synaptic GABA_A_R (8). Two anti-GABA_A_R encephalitis patients were reported and their autoantibodies targeted the β3 subunits (9). Later, a study identified the main antigens as α1/γ2 in a group of patients with seizures and cognitive or neuropsychiatric problems. Some of these patients had mood changes (2 in 11 showed depression symptoms and the autoantibodies targeted to α1 or undefined; 3 showed anxiety and the autoantibodies targeted to α1, γ2, or undefined subunits) (10). A cohort of purely depression disorders has not been tested so far.

#### Anti-GABA_B_ Receptor

GABA_B_ receptors are metabotropic transmembrane receptors that are linked to G-protein-gated potassium channels (119). There are two GABA_B_R subtypes, GABA_B1_R and GABA_B2_R, assembling into functional heterogenic complexes (120, 121). GABA_B1_R(−/−) mice, which lack functional GABA(B) receptors, showed more anxiety and decreased immobility (antidepressant-like behavior), and GABA_B_R selective antagonist CGP56433A showed antidepressant effects as well (122).

Autoantibodies to the GABA_B_R (anti-GABA_B_R) were reported in limbic encephalitis (15 in 410 cases) (7). In all patients, autoantibodies to GABA_B_R targeted the GABA_B1_R and only one targeted GABA_B2_R additionally (123, 124). If anti-GABA_B_R inactivates synaptic and extrasynaptic GABA_B_R, it could potentially cause anxiety but not depression. Additionally, one anti-GABA_B_R (B1/B2) positive patient was found in a depression cohort (*n* < 310) by fixed CBA using 1:10 diluted serum with all the controls being seronegative (*n* > 1,693) (102). To date, there are only limited studies that focus on this antigen and further investigations should be performed to extend the knowledge about GABA_B_R autoantibody effector mechanisms.

### Anti-Monoamine Receptor Autoantibodies

#### Anti-5-HT1A Receptor and anti-D2 Antibodies

The 5-HT1A receptor is a subtype of serotonin receptor expressed widely in the limbic system and has implications in the control of mood, cognition, and memory (125). D2R is a dopamine receptor and has long isoforms (located mainly on the postsynaptic membrane) and short isoforms (mainly on the presynaptic membrane), coded by alternative splicing of the same DRD2 gene (126). It is highly expressed in basal ganglia and also cortex, hippocampus, and in substantia nigra and is involved in synaptic plasticity and memory formation (127). Both receptors are coupled with G-proteins that inhibit adenylyl cyclase, as well as other second messenger cascades (125, 128).

The presence in serum of IgG autoantibodies against 5-HT1A (anti-5-HT1A) and dopamine receptor D_2_ (anti-D2R) in psychiatric disorders was studied by radioimmunoassay (RIA) (129). 7.9% of the mood disorder patients including 33 MDD had anti-5-HT1A and 9.5% had anti-D2R compared to healthy controls which were seronegative for these autoantibodies. Anti-D2R was significantly associated with the severity of guilt feeling and depressive mood. To our knowledge, no further experiments have been reported detecting or investigating the role of anti-5-HT1A in psychiatric disorders.

Immunoglobulin G autoantibodies against D2R were identified by flow cytometry CBA with a cutoff at three SDs above the control mean using transfected HEK cells in a subgroup of children with basal ganglia encephalitis (15). 12 of 17 children (aged 0.4–15 years, nine males) with basal ganglia encephalitis had anti-D2R, compared with 0 in 67 controls. The 12 anti-D2R-positive patients had movement disorders and psychiatric disturbance characterized by Parkinsonism, dystonia, chorea, emotional lability, attention deficit, and psychosis. A later study showed a specific and significant reduction of D2R when transfected cells were incubated with anti-D2R, and the extracellular N-terminus of D2R was revealed as the main immunogenic region (130). 3 anti-D2R-positive cases out of 43 were reported in first episode of acute psychosis in children and the 17 controls studied were seronegative (131). This is the first report of serum IgG autoantibodies to surface D2R in pediatric patients with isolated psychosis. And three of the patients were previously diagnosed with other types of mental disorders: one patient had attention-deficit/hyperactivity disorder, behavior disorder, one had depression and anxiety, prematurity, and one had anorexia nervosa (131).

### Anti-VGKC Complex and Associated Protein Autoantibodies

#### Anti-LGI1, Anti-Caspr2, and Anti-DPPX

Voltage-gated potassium channels, typically formed by four different α subunits (there are 40 α subunits known), each associated with a β subunit (more than 12 β auxiliary proteins to α subunits), play a crucial role in returning the depolarized cell such as neurons to a resting state (26, 132). Typically, they are tetramers of four α subunits arranged as a ring, each contributing to the wall of the transmembrane K+ pore. Additionally, there are other associated proteins like LGI1, Caspr2, contactin 2, ADAM22, and ADAM23, which can affect the function of VGKC and AMPAR (mentioned in the antibody effector function section) (133).

Autoantibodies to the VGKC complex (anti-VGKC complex) have been known for a long time and are involved in the pathogenesis of neuromyotonia, Morvan’s syndrome, epilepsy, and limbic encephalitis (26, 134, 135). In recent years, researchers identified by CBA and IHC that the VGKC-associated proteins LGI1 and Caspr2 are actually the main targets in autoimmune encephalitis. Kv4.2, a subtype of VGKC, is widely expressed in the CNS and autoantibodies directed against DPPX (an auxiliary subunit of Kv4.2 channels) (anti-DPPX) was also identified, yet in approximately 19% of the seropositive cases for the VGKC complex by RIA the antigen/s remain unknown (11, 14). Epilepsy and limbic encephalitis are more frequently related to anti-LGI1, while peripheral nerve hyperexcitability disorders, like Morvan’s syndrome, are more common in anti-Caspr2-positive cases (136). Anti-LGI1 patients present a clinical spectrum of confusion, depression, paranoia, behavior disturbances, visual hallucinations, and dementia at onset of the disease (137–139). Two seropositive (one IgG type) anti-Caspr2 were found in a cohort of 310 patients with affective disorders, while in the same study, no anti-LGI1 and anti-DPPX seropositive cases were reported (102). The largest described cohort of anti-DPPX (IgG)-positive patients consisted of 20 cases. Those sera or CSF-positive cases were found in patients referred for evaluation of paraneoplastic neurologic autoimmunity (totally tested 83) and 41,812 samples submitted for evaluation of neural autoantibodies (0.02% positive anti-DPPX). Out of the 20 anti-DPPX-positive patients, 20% showed depressive symptoms (14).

## Take-Home Message

Although an increasing number of studies have substantially improved our knowledge on autoimmunity in the CNS, still large controversy exists, especially due to the variation in the methodology used. Also, our knowledge is largely based on findings from autoimmune encephalitis cohorts. There are several methodological aspects which have to be considered when detecting NSAbs in psychiatric disorders, especially in depression or other mood disorders. First, the antigens targeted by the autoantibodies can be composed of several subunits. Autoantibodies against each of the subunits can have different clinical significance and implications (1). A good example is the detection of NMDA NR1 antibodies and N2A/B antibodies. Anti-NR1 is believed to be pathogenic in NMDAR encephalitis (97). However, anti-N2A/B plays a role in NPSLE (108). When autoantibodies target different subunits of other glutamate receptors or GABA receptors, they may cause different clinical symptoms. At the same time, most NSAbs target epitopes only if the antigens are expressed in their native conformation. Techniques like CBA, IHC of brain sections optimized to detect membrane proteins (rodent), and immunocytochemistry of cultures of rodent live hippocampal neurons fit this requirement. Third, different concentrations of the same autoantibody might have different effects and biological relevance. For example, high titers of anti-GABA_A_R are specific for severe encephalitis and refractory seizures patients and low titers present in a broad range of neurology disorders and may lack specificity (8). Another aspect which needs to be taken into account is the value of serum and CSF for detecting autoantibodies. The use of CSF for detecting NSAbs in depression has not been evaluated to date. Finally, NSAbs should be tested in a “panel”, rather than a single one because of the overlap between symptoms and signs of different autoimmune encephalitis and psychotic disorders (140). Also, the coexistence of several NSAbs may occur in individual patients and cause combined manifestations (9, 141, 142).

To summarize, NSAbs, targeting important neuronal receptors or interfering with ion channels and associated protein function, are responsible for psychiatric symptoms in autoimmune encephalitis cases. At the moment, several studies reported the presence of anti-NMDAR (NR1 and NR2B), anti-5-HT1A, and D2R in depression cohorts. However, due to the heterogeneity of the methodology, variation in the samples used, and the limited cohort size, there is insufficient evidence to support those NSAbs can cause depression without other obvious neurological symptoms. In the future, large cohorts, longitudinal studies need to be performed using sensitive, quantitative, and reproducible methods without loss of antigen conformation. Finally, analysis of autoantibodies targeting neuronal surface antigens relevant to the pathology of depression should be performed.

## Author Contributions

SZ contributes in the design, writing, and correcting of the paper. CH and MD contributed the writing and corrections; PM helped with the corrections of the review; ML helped with design and the corrections of the review; and PM-M supervised the design and helped to write and correct the review.

## Conflict of Interest Statement

The authors declare that the research was conducted in the absence of any commercial or financial relationships that could be construed as a potential conflict of interest.
